# Meta-analysis of neoadjuvant chemotherapy versus neoadjuvant chemoradiotherapy for locally advanced rectal cancer

**DOI:** 10.1186/s12957-021-02251-0

**Published:** 2021-05-05

**Authors:** Huaqin Lin, Lei Wang, Xiaohong Zhong, Xueqing Zhang, Lingdong Shao, Junxin Wu

**Affiliations:** Department of Radiation Oncology, Fujian Medical University Cancer Hospital, Fujian Cancer Hospital, Fuzhou, China

**Keywords:** Locally advanced rectal cancer, Neoadjuvant chemoradiotherapy, Neoadjuvant chemotherapy, Meta-analysis

## Abstract

**Background and purpose:**

With the advent of more intensive chemotherapy regimens, neoadjuvant chemoradiotherapy (NACRT) for patients with locally advanced rectal cancer (LARC) has always been questioned due to its inevitable radiation toxicity. Hence, we conducted a meta-analysis to compare the clinical efficacy of neoadjuvant chemotherapy (NAC) and NACRT.

**Materials and methods:**

Eligible studies were searched using PubMed, MEDLINE, Embase, the Cochrane Library, and Web of Science up to 31 July 2020, comparing the clinical efficacy of NAC versus NACRT for LARC. Short- and long-term outcomes were determined using the odds ratio (OR) with 95% confidence interval (CI).

**Results:**

Six studies with 12,812 patients were eligible for this meta-analysis, including 677 patients in the NAC group and 12,135 patients in the NACRT group. There were no significant differences between the two groups in terms of pathological complete response rate (OR=0.62, 95%CI=0.27~1.41), N down-staging rate (OR=1.20, 95%CI=0.25~5.79), R0 resection rate (OR=1.24, 95%CI=0.78~1.98), and local relapse rate (OR=1.12, 95%CI=0.58~2.14). The pooled OR for the total response rate and T down-staging were in favor of NACRT (OR=0.41, 95%CI=0.22~0.76 versus OR=0.67 95%CI=0.52~0.87). However, the pooled OR for the sphincter preservation rate favored NAC compared with NACRT (OR=1.87, 95%CI=1.24~2.81). Moreover, NAC was found to be superior to NACRT in terms of distant metastasis (14.3% vs. 20.4%), but the difference was not significant (OR=0.84, 95%CI=0.31~2.27).

**Conclusion:**

We concluded that NAC was superior to NACRT in terms of the sphincter preservation rate, and non-inferior to NACRT in terms of pCR, N down-staging, R0 resection, local relapse, and distant metastasis. However, the conclusion warrants further validation.

**Supplementary Information:**

The online version contains supplementary material available at 10.1186/s12957-021-02251-0.

## Introduction

Colorectal cancer (CRC) is the third leading cancer worldwide. Approximately eight hundred and sixty thousand patients die of CRC annually [1]. Patients with rectal cancer typically have a better prognosis than those with colon cancer [1], but various kinds of neoadjuvant modalities have been tried to improve the prognosis of patients with rectal cancer, especially for those with locally advanced rectal cancer (LARC). Among the current neoadjuvant modalities, neoadjuvant chemoradiotherapy (NACRT) is preferred [2, 3].

However, the clinical value of NACRT has always been questioned. Data from the Surveillance, Epidemiology, and End Results (SEER) showed that less than 37.5% of patients received NACRT [4]. Reasons are as follows: (1) the survival benefit of NACRT has not been confirmed up to today [5–9]; (2) the current rate of pathological complete response following NACRT is 10% to 25% [10, 11], which is far from satisfactory; (3) adverse events (AEs) related to radiotherapy including radiation colitis decrease the compliance of patients [12–14]; and (4) radiotherapy is deemed to increase the difficulty of surgical dissection and the risk of postoperative complications, which often make the surgeons hesitate to applicate it.

In recent decades, intensive regimens of adjuvant chemotherapy including the FOFLOX [15] and fluorouracil and leucovorin and oxaliplatin regimens [16] have been confirmed as superior to conventional regimens. This highlights the potential use of neoadjuvant chemotherapy (NAC) alone. Matsumoto et al. [17] found that NAC could acquire a similar rate of pathological complete response (pCR) but without radiation toxicity, which was confirmed by the latter reports [18]. In addition, NAC was also found to increase the rate of sphincter preservation by Okuyama et al. [18]. Considering that the results of published studies were not consistent, we conducted a meta-analysis to compare the clinical efficacy of NAC and NACRT for LARC.

## Methods

This systematic review was performed according to the guidelines of the Preferred Reporting Items for Systematic Reviews and Meta-Analyses (PRISMA) [19] and Assessing the methodological quality of systematic reviews (AMSTAR) Guidelines [20].

### Literature search

PubMed, MEDLINE, Embase, the Cochrane Library, and Google Scholar were used to identify the potentially eligible studies comparing the clinical efficacy of NAC and NACRT in patients with LARC, up to 31 July 2020, by two independent researchers from the same establishment. The keywords “rectal cancer,” “adenocarcinoma of rectum,” “local staging,” “locally advanced rectal cancer,” “neo-adjuvant,” “chemotherapy,” “neo-adjuvant chemotherapy,” “Concomitant Chemoradiotherapies,” “surgery,” and “Neoadjuvant Treatment” were used in all possible combinations. Studies comparing the clinical efficacy of NAC and NACRT for patient with LARC. Search strategy for MEDLINE via PubMed was depicted in Supplement 1. Searching strategies for other databases were performed correspondingly. In addition, relevant trials either ongoing or unpublished were also searched in www.clinicaltrials.gov and www.clinicaltrialsregister.eu. Any potentially eligible studies were searched manually from the included studies, reviews, letters, comments, and abstracts from meetings.

### Selection criteria

Inclusion criteria are as follows: (1) patients were diagnosed as rectal adenocarcinoma by biopsy specimen; (2) patients were staged at T_any_N_+_ M_0_ or T_3/4_N_any_ M_0_ using CT/MRI/rectal ultrasound; (3) intervention included NAC and NACRT; and (4) outcomes including rates of pCR, response, T down-staging, N down-staging, R0 resection, sphincter preservation, local relapse, distant metastasis, and complications.

Exclusion criteria are as follows: (1) patients with colorectal cancer; (2) tumors were unresectable at diagnosis; (3) single arm studies; (4) case reports, letters, and reviews; (5) follow-up information is incomplete; and (6) pCR data is unknown.

Considering short- and long-term outcomes of most of the trials were reported separately, all the publications of each trial including conference abstracts were identified.

### Data extraction

All data were extracted and assessed by two independent investigators with predefined forms, which were as follows: (1) general data including title, first author, journal, publication data, and study design; (2) baseline characteristics, such as tumor stage, patient number, chemoradiotherapy regimens, surgical modality, adjuvant regimens, neoadjuvant chemoradiotherapy, and neoadjuvant chemotherapy; (3) outcomes including short- and long-term outcomes (pCR, response, T down-staging, N down-staging, R0 resection, sphincter preservation, local relapse, and distant metastasis). In the case of disagreement, a third investigator intervened for a conclusion. Considering that pCR is the most widely used surrogate indicator of preoperative neoadjuvant therapy, we took it as the primary and others as secondary outcomes in this meta-analysis.

### Definition of outcomes

pCR was defined as absence of microscopic adenocarcinoma cells in the surgical specimen [21].

Local relapse was defined as evidence of tumor within the pelvic or perineal area.

Distant metastasis was defined as recurrence outside the true pelvis.

R0 resection was defined as no evidence of tumor at the surgical margin macroscopically or pathologically.

T-down staging was defined as the reduction of pathological T stage (ypT) from clinical T stage.

N-down staging was defined as the reduction of pathological N stage (ypN) from clinical N stage.

Overall survival (OS) was defined as time to death from any cause, or to end of follow-up (censored).

Disease-free survival (DFS) was defined as time to any recurrence or death, whichever occurred first, or end of follow-up (censored).

### Quality assessment

The quality of each included study was determined according to the modified Newcastle-Ottawa Scale (NOS) [22], which contained the three following parts with full score of 9: the selection of study groups (0–4 points), the comparability between the two groups (0–2 points), and the determination of either the exposure or the outcome of interest (0–3 points). Generally, studies scored above 5 were considered to be of high quality.

### Statistical analysis

The systematic review and meta-analysis were registered at https://www.crd.york.ac.uk/PROSPERO/ (Review Registry 213733) and performed using RevMan version 5.3 and Stata 15. Odds ratio (OR) with its 95% confidence interval (CI) was chosen as an effect measure to evaluate the rates of pCR, response, T down-staging, N down-staging, R0 resection, sphincter preservation, local relapse and distant metastasis. All results would be investigated for statistical heterogeneity by *I*^2^ statistics. If there is considerable heterogeneity (*I*^2^ > 50%, *P*< 0.05) for an outcome, random-effect model would be used [23]. Nevertheless, a sensitivity analysis would be performed in each outcome by removing one of the included studies at a time. Forest plots were conducted to evaluate the publication bias with Begg’s [24] and Egger’s test [25].

## Results

### Basic characteristic of the included studies

**Fig. 1:**
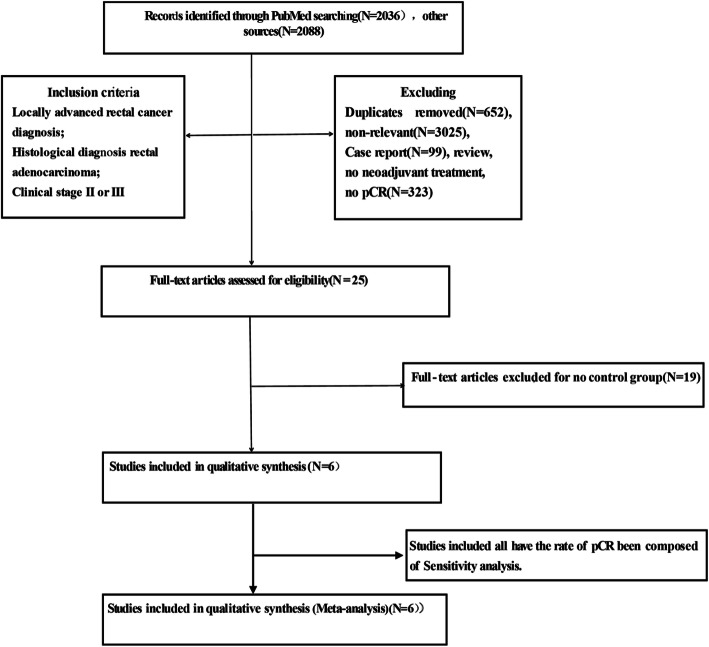
PRISMA flow diagram showing a selection of articles for meta-analysis

According to the predesigned searching strategy, 4124 records were identified by two independent reviewers, and then 4118 records were excluded based on the established inclusion and exclusion criteria. Finally, seven trials remained to be analyzed in this study (Fig. 1).

Totally, 12,812 patients were enrolled in this meta-analysis, including 677 patients in the NAC group, 12,135 patients in the NACRT group, respectively. Characteristics of each trial and score of each included study were depicted in Table 1. Briefly, there is only one randomized controlled trial (RCT) [29] eligible for this meta-analysis, and four of the six included studies came from Japan [17, 18, 26, 28]. Patients from China cohorts were much younger than those from other cohorts and had a higher proportion of T4 [29]. Of note, one [27] of the included studies was scored as five, one [18] as six, three [17, 26, 29] as seven, and one [28] as eight, respectively. In addition, the score of each item was depicted in Supplement Table 1.

**Table 1:** Characteristics and treatments of the included studies

Author/year	Country	Arm	No. of Pts	Treatment regimens	Type of surgery	Duration	Design	Median age (years)	Clinical T category (T2/T3/T4)	Clinical N category (N0/N1/N2/N3)	Clinical stage (I/II/III/IV)	Follow-up (months)	pCR	R0	NOS
Matsumoto, 2015 [17]	Japan	NAC	15	FOLFOX: 6 cycles, IRIS: 3 cycles, FOLFIRI: 5 cycles	LAR (12), APR/ASR (2), ISR (1)	2005–2010	Retrospective	64 (56–68)	0/12/3	NA	NA	44.2 (30.7–59.5)	2	15	7
		NACRT	109	NA	LAR (75), APR/ASR (22), ISR (3)			67 (61–74)	0/92/17	NA	NA		0	105	
Sakuyama, 2016 [26]	Japan	NAC	44	FOLFOX: 6 cycles	ISR (34), other (10)	2001–2014	Retrospective	57.4 (28–76)	0/38/6	5/16/7/16	0/5/35/4	NA	4	NA	7
		NACRT	44	5FU+RT (45 Gy/25 F)	ISR (44)			56 (27–77)	9/35/0	27/10/6/1	6/19/29/0		9	NA	
Okuyama, 2018 [18]	Japan	NAC	27	SOX+cetuximab, SOX+mFOLFOX6	LAR (19), APR (8)	2010–2016	Retrospective	66 (40–79)	0/24/3	0/18/9/0	NA	45.4	1	26	6
		NACRT	28	5FU+RT (45 Gy/25 F)	LAR (8), APR (20)			68 (42–78)	0/22/5	0/17/11/0	NA		4	26	
Sada, 2018 [27]	American	NAC	410	NA	NA	2006–2010	Retrospective	NA	NA	NA	NA	NA	35	NA	5
		NACRT	11614	NA	NA			NA	NA	NA	NA		1352	NA	
Sato, 2019 [28]	Japan	NAC	16	SOX:S1+Ox: 3 cycles	Laparoscopically	2002–2016	Retrospective	67.5 (43–77)	NA	NA	NA	NA	2	NA	8
		NACRT	10	5FU+RT (40–45Gy)	Laparotomy			66 (53–71)	NA	NA	NA		0	NA	
Deng, 2019 [29]	China	NAC	165	mFOLFOX6	NA	2010–2015	RCT	54.1	1/114/50	46/76/43/0	0/46/119/0	NA	10	136	7
		NACRT	330	5FU/mFOLFOX6+RT (46–50.4 Gy/23–25 F)	NA			54.1/52.1	11/206/113	67/172/91	0/67/263/0		61	262	

*Note*: *No. of Pts* Number of patients, *RCT* randomized controlled trial, *pCR* pathologic complete response, *NAC* neoadjuvant chemotherapy without radiation, *NACRT* neoadjuvant chemoradiotherapy, *5FU* 5-fluorauracil, *OX* oxaliplatin, *RT* radiotherapy, *FOLFOX* fluorouracil, leucovorin, and oxaliplatin, *IRIS* irinotecan, tegafurgimeracil-oteracil potassium, *FOLFIRI* fluorouracil, leucovorin, and irinotecan, *SOX* S-1+ oxaliplatin, *S-1* tegafurgimeracil-oteracil potassium, *mFOLFOX6* modified infusional fluorouracil, leucovorin, and oxaliplatinm, *XELOX* capecitabine + oxaliplatinm, *APR*, abdominoperineal resection, *ISR* intersphincteric resection, *LAR* low anterior resection, *ASR* abdominosacral resection, *NOS* Newcastle-Ottawa Scale, *NA* not available

Regimens of NAC and NACRT in each included study were depicted in Table 2, which showed that differences existed among the different studies, especially for the regimen of NAC. Surgical techniques were also depicted in Table 2.

**Table 2:** Outcomes of included studies

Outcomes	Terms	HR (95%CI)	Heterogenicity	Heterogenicity	Effect size	Effect size^**2**^
			***P***	***I***^**2**^ (%)	***Z***	***P***
pCR	6	0.62 (0.27, 1.41)	0.01	66	1.15	0.25
R0 Resection	3	1.24 (0.78, 1.98)	0.93	0	0.91	0.36
sphincter preservation	5	1.87 (1.24, 2.81)	0.20	35	3.01	0.003
Response	4	0.46 (0.27, 0.76)	0.02	68	3.02	0.003
N downstaging rates	3	1.20 (0.25, 5.79)	<0.001	90	0.23	0.82
T downstaging rates	3	0.67 (0.52, 0.87)	0.36	2	2.99	0.003
Local relapse rate	3	1.12 (0.58, 2.14)	0.86	0	0.33	0.74
Distant metastases rate	2	0.84 (0.31, 2.27)	0.23	31	0.35	0.73

Note: *pCR* pathologic complete response, *HR (95%CI)*, hazard rate (95% confidence interval)

### Meta-analysis of tumor response to neoadjuvant treatment

**Fig. 2:**
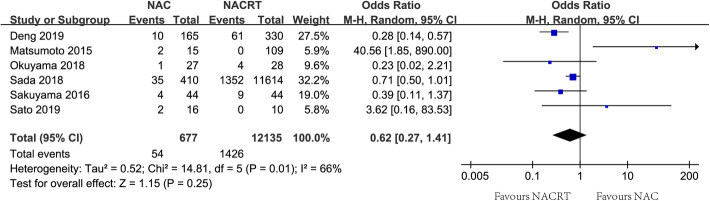
Forest plot of pathological complete response rate between groups of NAC and NACRT

pCR as the primary endpoint was evaluated in six included studies [17, 18, 26–29], and a significant difference was observed among the included studies (*I*^2^=66%, *P*=0.01). The rate of pCR was lower in the NAC group than that in the NACRT group (8.0% vs. 11.8%), but there was no significant difference between groups of NAC and NACRT using a random-effect model (OR=0.62, 95%CI=0.27~1.41, Fig. 2). The rates of total response, T down-staging, and N down-staging were evaluated in the included studies of four [18, 26, 27, 29], three [26–28], and three [26–28], respectively. *I*^2^ for the meta-analysis of total response, T down-staging and N down-staging were 68% (*P*=0.02), 2% (*P*=0.36), and 90% (*P*< 0.01), respectively. The pooled OR for the rate of response was not in favor of NAC using a random-effect model (31.6% vs. 42.5%, OR=0.63, 95%CI=0.46~0.85, Table 2). The inferiority of NAC to NACRT was also found in the pooled OR for the rate of T down-staging using a fixed-effect model (16.4% vs. 20.1%, OR=0.67, 95%CI=0.52~0.87, Table 2), but there was no significant difference in the pooled OR for the rate of N down-staging (46.6% vs. 56.3%, OR=1.20, 95%CI=0.25~5.79, Table 2).

### Meta-analysis of R0 resection and sphincter preservation

**Fig. 3:**
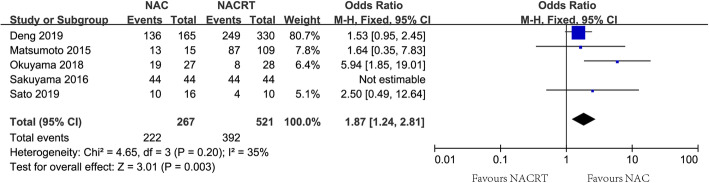
Forest plot of sphincter preservation between groups of NAC and NACRT

The rate of R0 resection was evaluated in three included studies [17, 18, 29], and no heterogeneity was found (*I*^2^=0, *P*=0.93). Using a fixed-effect model, there was no significant difference in the pooled rate of R0 resection between groups of NAC and NACRT (85.5% vs. 84.2%, OR=1.24, 95%CI=0.78~1.98, Table 2). The rate of sphincter preservation as the secondary outcome was evaluated in five included studies [17, 18, 26, 28, 29], and significant heterogeneity was not observed among the included studies (*I*^2^=35%, *P*=0.20). The pooled OR for the rate of preservation was in favor of NAC using a fixed-effect model (83.1% vs. 75.2%, OR=1.87, 95%CI=1.24~2.81, Fig. 3).

### Meta-analysis of local relapse and distant metastasis

The rate of local relapse was evaluated in four included studies [17, 18, 29], and no heterogeneity was found among the included studies (*I*^2^=0, *P*=0.86). The rate of local relapse was comparable between groups of NAC and NACRT (7.2% vs. 6.4%), and the pooled OR was 1.12 (95%CI=0.58~2.14, Table 2) using a fixed-effect model. The rate of distant metastasis was evaluated in two included studies [17, 18], and significant heterogeneity was not found among the included studies (*I*^2^=31%, *P*=0.23). The rate of distant metastasis was lower in the NAC group than that in the NACRT group (14.3% vs. 20.4%), but the pooled OR for the rate of distant metastasis was 0.84 (95%CI=0.31~2.27, Table 2) using a fixed-effect model.

### Complications

Complications were evaluated in three included studies, which were depicted in Table 3. Generally, there was no significant difference in each included study.

**Table 3:** Complications of the included studies

Study	Treatment	Patients	Related complications
Matsumoto, 2015 [17]	NAC	15	Grade 3–4 adverse events: 2 neutropenia and 1 diarrhea
	NACRT	109	NA
Okuyama, 2018 [18]	NAC	27	Grade 3–4 adverse events: no; Frequent toxic events:grade 1 neutropenia
	NACRT	28	Grade 3–4 adverse events: no; Frequent toxic events: grade 1 diarrhea
Deng, 2019 [29]	NAC	152	Grade 3/4 toxicities: leukopenia 9; neutropenia 15; nausea/vomiting 4; diarrhea 12
	NACRT	292	Grade 3/4 toxicities: leukopenia 48; neutropenia 40; nausea/vomiting 13; diarrhea 35; radiation dermatitis 54; radiation proctitis 35;

Note: *NAC* neoadjuvant chemotherapy without radiation, *NACRT* neoadjuvant chemoradiation therap, *NA* not available

### Sensitivity analysis

**Fig. 4:**
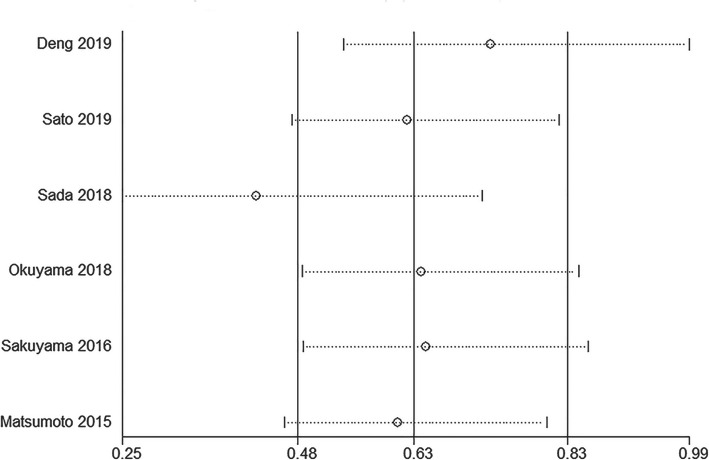
Sensitivity analysis of the pathological complete response

Sensitivity analysis was conducted for the rate of pCR. Result showed that the pooled OR would not change greatly by removing any single included study (Fig. 4), which indicated that the result was robust.

### Publication bias

**Fig. 5:**
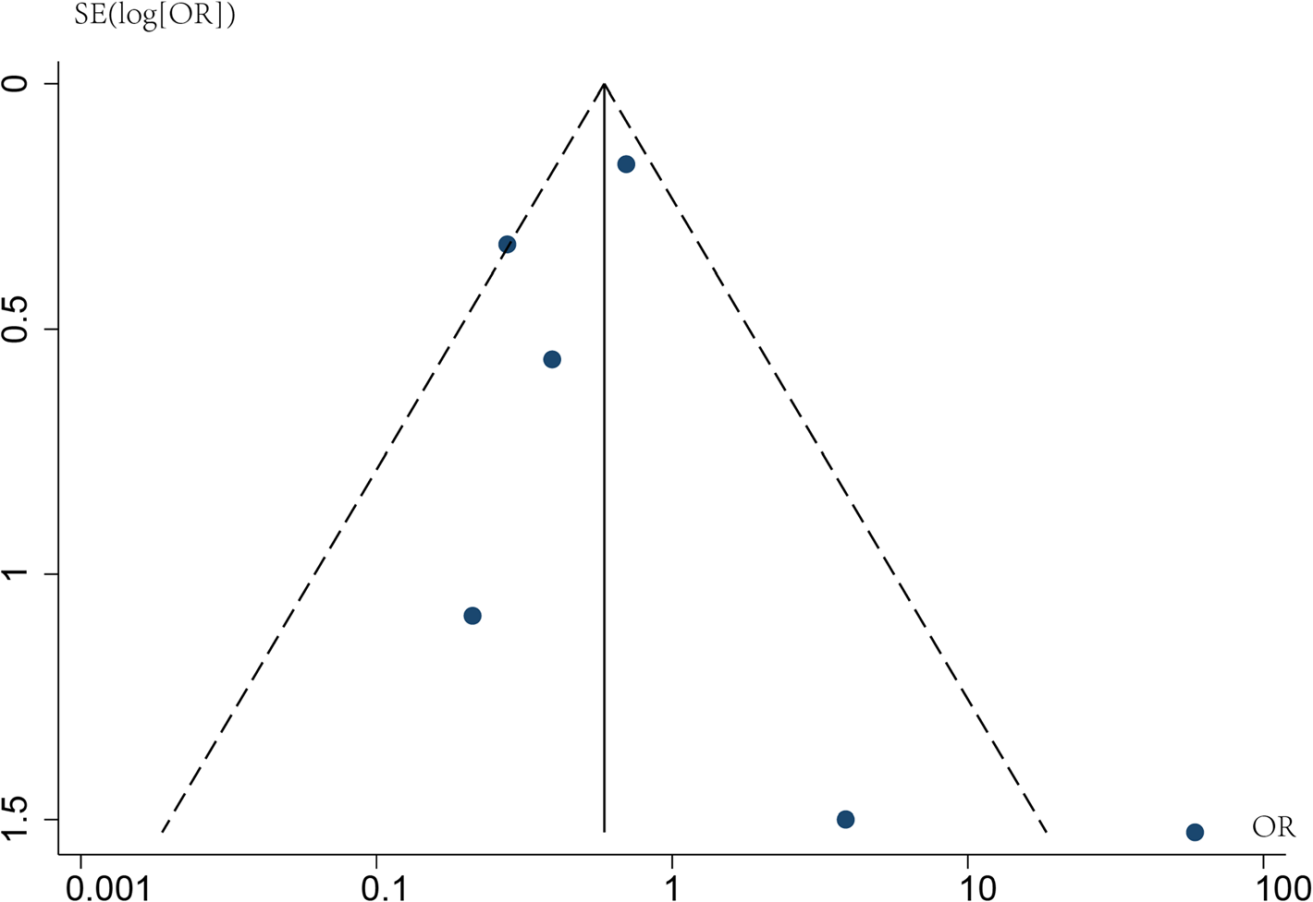
Funnel plot about publication bias of pathological complete response

Publication bias was evaluated for the rate of pCR. Asymmetry was not observed in the funnel plot for the rate of pCR (Fig. 5), and the *P* value of Egger’s and Begg’s tests were 0.452 and 0.568, respectively.

## Discussion

NACRT followed by surgery has been the standard treatment for LARC according to the guidelines of the National Comprehensive Cancer Network (NCCN) [2], but its long-term efficacy including both OS and DFS has never been confirmed [5, 6]. On the other hand, previous clinical trials have identified the clinical efficacy of NAC alone [30–43] which challenged the current neoadjuvant modality. This was the first meta-analysis comparing the clinical efficacy of NAC and NACRT for patients with LARC. Six studies including 12,812 patients were identified to be eligible in this meta-analysis. Results showed that NAC was not inferior to NACRT in terms of pCR, R0 resection, local relapse, and distant metastasis, but was superior to NACRT in terms of sphincter preservation.

pCR is one of the most important indexes to evaluate the efficacy of neoadjuvant treatments. Compared with neoadjuvant radiotherapy or chemotherapy alone, NACRT has a higher rate of pCR [44], although it remains unsatisfactory. But no significant difference was observed in terms of pCR between groups of NAC and NACRT in this meta-analysis (8.0% vs. 11.8%, OR=0.62, 95%CI=0.27~1.41), mainly because more intensive chemotherapy regimens and more courses were administrated in the group of NAC. Nonetheless, the advantage of NACRT over NAC in terms of the total response rate and T down-staging was confirmed in this meta-analysis (16.4% vs. 20.1%, OR=0.67, 95%CI=0.52~0.87). N down-staging is an advantage of NAC, but in this meta-analysis, there was no significant difference between groups of NAC and NACRT. Hence, we recommend NACRT as the first-line treatment for LARC patients, if they are present with a more advanced T stage or unwilling to receive surgery.

Sphincter preservation is well concerned by surgeons and patients, especially for those with lower LARC. Sphincter preservation is also one of the initial aims of neoadjuvant treatments, but NARCT was reported to be associated with an increased risk of fistula and worse anal function [12, 45]. In this meta-analysis, NAC is found to be superior to NACRT in terms of sphincter preservation (83.1% vs. 75.2%, OR=1.87, 95%CI=1.24~2.81). Hence, NAC would be recommended first for patients with a strong willingness to preserve anal.

In the era of total mesorectal excision (TME), local relapse is no longer fatal with the rate of 11% [5, 46]. In this meta-analysis, NAC exhibited equivalent efficacy in terms of local relapse (7.2% vs. 6.4%). On the contrary, distant metastasis is still the dominant cause for treatment failure, which is reported to be as high as 25% to 30% [6, 47, 48]. In this meta-analysis, we found that the rate of distant metastasis was lower in the NAC group than that in the NACRT group (14.3% vs. 20.4%), although it lacked statistical significance in the pooled OR (OR=0.84, 95%CI=0.31~2.27). Considering that distant metastasis was only evaluated in two included studies and both of them were from Japan [17, 18], where lateral lymph node dissection (LLND) was conducted routinely in the procedure of surgery, subgroup analysis stratified by LLND or not should be expected in future.

The advantages of NAC also lie on its low toxicity and high compliance. Generally, NAC but not NACRT could not cause severe fibrosis, which often increases the difficulty of surgery and risk of the fistula. Radiotherapy toxicity such as radiation colitis is inevitable, although most of the radiation colitis is mild and transient [49–51]. In addition, patients receiving NAC are more likely to receive adjuvant chemotherapy, compared with those receiving NACRT [52–54]. What is important, NAC could offer a chance for patients with early local recurrence to receive a salvage curative radiotherapy.

However, there were several limitations in this meta-analysis. First, most of the studies (5/6) were retrospective, which indicated that recalling bias and selection bias were hard to avoid. Second, in this meta-analysis, we included studies from 2011 to 2019, during which staging systems on rectal cancer have experienced a few changes on N staging [2]. According to the 8th (American Joint Committee on Cancer (AJCC) staging system, tumor deposits are defined as N1c if no regional lymph nodes are positive; while it was not referred in the 7th AJCC staging system. However, it might not bring substantial changes to our research, because LARC include T_any_ N_+_ or T_3/4_N_any_. Third, most of the studies (4/6) were from Japan, where LLND was conducted routinely, which would weaken the conclusion of this meta-analysis. Forth, the NAC regimens were largely different for each included study, and the optimum regimen has not been reached. The last but not the least, data on the adjuvant radiotherapy was not available, which would weaken the conclusion of this meta-analysis, although adjuvant radiotherapy is not routinely used in clinic.

## Conclusion

With the current data, we concluded that NAC could be taken as a reasonable alternative to CRT in LARC, and it should be given priority to recommend for patients with T2/3, and strong willingness to sphincter preservation. Nonetheless, NACRT should be recommended firstly if patients were present with T4, high-risk factors including positive circumferential margin involvement, and were not prepared to perform surgery. In future, more intensive NAC regimens including targeted drugs and/or immune therapies are expected, and identifying patients who would be benefited from each of the neoadjuvant modalities is the key.

## Supplementary Information

**Additional file 1: Table S1** Assessment of methodological quality of included studies for meta-analysis based on the Newcastle-Ottawa Scale for cohort studies.

**Additional file 2: Table S2** Search strategy in PubMed.

## Data Availability

All the data for this article can be found on PubMed, MEDLINE, Embase, the Cochrane Library, and Web of Science.

## Abbreviations

NACRT: Neoadjuvant chemoradiotherapy
LARC: Locally advanced rectal cancer
NAC: Neoadjuvant chemotherapy
OR: Odds ratio
CI: Confidence interval
CRT: Concurrent chemoradiotherapy
CRC: Colorectal cancer
SEER: Surveillance, Epidemiology, and End Results
AEs: Adverse events
FOFLOX: Oxaliplatin, fluorouracil (5-FU), and leucovorin
PCR: Pathological complete response
OS: Overall survival
DFS: Disease-free survival
NOS: Newcastle-Ottawa Scale
RCT: Randomized controlled trial
NCCN: National Comprehensive Cancer Network
TME: Total mesorectal excision
LLND: Lateral lymph node dissection
AJCC: American Joint Committee on Cancer
IRIS: Irinotecan, tegafurgimeracil-oteracil potassium
FOLFIRI: Fluorouracil, leucovorin and irinotecan
SOX: Oxaliplatin and tegafurgimeracil-oteracil potassium
mFOLFOX6: Modified infusional fluorouracil, leucovorin, and oxaliplatin
XELOX: Capecitabine oxaliplatin
APR: Abdominoperineal resection
ISR: Intersphincteric resection
LAR: Low anterior resection
ASR: Abdominosacral resection
