# Supplementary material for: Meta-analysis of neoadjuvant chemotherapy versus neoadjuvant chemoradiotherapy for locally advanced rectal cancer
Source: World J Surg Oncol. 2021 May 5;19:141. doi: 10.1186/s12957-021-02251-0 (PMC8101236; doi:10.1186/s12957-021-02251-0)
Supplement: Supplementary file 1 — Additional file 1: Table S1 Assessment of methodological quality of included studies for meta-analysis based on the Newcastle-Ottawa Scale for cohort studies. [file 12957_2021_2251_MOESM1_ESM.docx]

Supplement Table 1 Assessment of methodological quality of included studies for meta-analysis based on the Newcastle-Ottawa Scale for cohort studies.

| Study | Selection | Comparability | Outcome/exposure | Total (max 9) |
| --- | --- | --- | --- | --- |
| Matsumoto 2015 | ★★★ | ★★ | ★★ | 7 |
| Sakuyama 2016 | ★★ | ★★ | ★★★ | 7 |
| Okuyama2018 | ★★ | ★★ | ★★ | 6 |
| Sada 2018 | ★★ | ★ | ★★ | 5 |
| Sato2019 | ★★★ | ★★ | ★★★ | 8 |
| Deng 2019 | ★★ | ★★ | ★★★ | 7 |
