# Supplementary material for: Meta-analysis of neoadjuvant chemotherapy versus neoadjuvant chemoradiotherapy for locally advanced rectal cancer
Source: World J Surg Oncol. 2021 May 5;19:141. doi: 10.1186/s12957-021-02251-0 (PMC8101236; doi:10.1186/s12957-021-02251-0)
Supplement: Supplementary file 2 — Additional file 2: Table S2 Search strategy in PubMed. [file 12957_2021_2251_MOESM2_ESM.docx]

Supplement Table 2 Search strategy in PubMed

| #1 | ("Rectal Neoplasms"[Mesh]) OR (Neoplasm, Rectal[Title/Abstract]) OR (Rectal Neoplasm[Title/Abstract]) OR (Rectum Neoplasms[Title/Abstract]) OR (Neoplasm, Rectum[Title/Abstract]) OR (Rectum Neoplasm[Title/Abstract]) OR (Rectal Tumors[Title/Abstract]) OR (Rectal Tumor[Title/Abstract]) OR (Tumor, Rectal[Title/Abstract]) OR (Neoplasms, Rectal[Title/Abstract]) OR (Cancer of Rectum[Title/Abstract]) OR (Rectum Cancers[Title/Abstract]) OR (Rectal Cancer[Title/Abstract]) OR (Cancer, Rectal[Title/Abstract]) OR (Rectal Cancers[Title/Abstract]) OR (Rectum Cancer[Title/Abstract]) OR (Cancer, Rectum[Title/Abstract]) OR (Cancer of the Rectum[Title/Abstract]) |
| --- | --- |
| #2 | ("Neoadjuvant Therapy"[Mesh]) OR (Neoadjuvant Therapies) OR (Therapies, Neoadjuvant) OR (Therapy, Neoadjuvant) OR (Neoadjuvant Treatment) OR (Neoadjuvant Treatments) OR (Treatment, Neoadjuvant) OR (Treatments, Neoadjuvant) |
| #3 | ("Drug Therapy"[Mesh]) OR (Therapy, Drug[Title/Abstract]) OR (Drug Therapies[Title/Abstract]) OR (Therapies, Drug[Title/Abstract]) OR (Chemotherapy[Title/Abstract]) OR (Chemotherapies[Title/Abstract]) OR (Pharmacotherapy[Title/Abstract]) OR (Pharmacotherapies[Title/Abstract]) |
| #4 | ("Chemoradiotherapy"[Mesh]) OR (Chemoradiotherapies[Title/Abstract]) OR (Radiochemotherapy[Title/Abstract]) OR (Radiochemotherapies[Title/Abstract]) OR (Concurrent Chemoradiotherapy[Title/Abstract]) OR (Chemoradiotherapies, Concurrent[Title/Abstract]) OR (Chemoradiotherapy, Concurrent[Title/Abstract]) OR (Concurrent Chemoradiotherapies[Title/Abstract]) OR (Synchronous Chemoradiotherapy[Title/Abstract]) OR (Chemoradiotherapies, Synchronous[Title/Abstract]) OR (Chemoradiotherapy, Synchronous[Title/Abstract]) OR (Synchronous Chemoradiotherapies[Title/Abstract]) OR (Concurrent Radiochemotherapy[Title/Abstract]) OR (Concurrent Radiochemotherapies[Title/Abstract])) OR (Radiochemotherapies, Concurrent[Title/Abstract])) OR (Radiochemotherapy, Concurrent[Title/Abstract])) OR (Concomitant Chemoradiotherapy[Title/Abstract])) OR (Chemoradiotherapies, Concomitant[Title/Abstract])) OR (Chemoradiotherapy, Concomitant[Title/Abstract]) OR (Concomitant Chemoradiotherapies[Title/Abstract]) OR (Concomitant Radiochemotherapy[Title/Abstract]) OR (Concomitant Radiochemotherapies[Title/Abstract]) OR (Radiochemotherapies, Concomitant[Title/Abstract]) OR (Radiochemotherapy, Concomitant[Title/Abstract]) |
| #5 | #1 and #2 and #3 and #4 |
